# The genetic diversity and population structure of domestic *Aedes aegypti* (Diptera: Culicidae) in Yunnan Province, southwestern China

**DOI:** 10.1186/s13071-017-2213-6

**Published:** 2017-06-13

**Authors:** Qing-Ming Shi, Heng-Duan Zhang, Gang Wang, Xiao-Xia Guo, Dan Xing, Yan-De Dong, Li Xiao, Jian Gao, Qin-Mei Liu, Ai-Juan Sun, Chun-Xiao Li, Tong-Yan Zhao

**Affiliations:** 1 State Key Laboratory of Pathogen and Biosecurity, Beijing Institute of Microbiology and Epidemiology, Beijing, Fengtai District China; 2 Center for Disease Control and Prevention of Chengdu Military Command, Chengdu, Jingjiang District China; 3 Zhejiang Entry-exit Inspection and Quarantine Bureau, Hangzhou, China; 4 Chengdu Medical College, Chengdu, Xindu District China

**Keywords:** *Aedes aegypti*, Genetic diversity, Population structure, Microsatellite loci, Genetic differences, Yunnan Province

## Abstract

**Background:**

There was no record of *Aedes aegypti* in Yunnan Province, China, until 2002, but this species is now continuously found in nine cities (or counties). Until now, little was known about the genetic diversity and population structure of this invasive species. Thus, a detailed understanding of the invasion strategies, colonisation and dispersal of this mosquito from a population genetics perspective is urgently needed for controlling and eliminating this disease vector.

**Methods:**

The genetic diversity and population structure of *Ae. aegypti* communities were analysed by screening nine microsatellite loci from 833 *Ae. aegypti* mosquitoes sampled from 28 locations in Yunnan Province.

**Results:**

In total, 114 alleles were obtained, and the average polymorphic information content (PIC) value was 0.672. The value of the alleles per locus ranged from 2.90 to 5.18, with an average of 4.04. The value of He ranged from 0.353 to 0.681, and the value of Ho within populations ranged from 0.401 to 0.689. Of the 28 locations, two showed significant departures from the Hardy-Weinberg equilibrium (HWE) with *P*-values less than 0.05, and a bottleneck effect was detected among locations from Ruili and the border areas with the degree of 60% and 50%, respectively. Combined with the F-statistics (F_IT_ = 0.222; F_CT_ = 0.145), the analysis of molecular variance (AMOVA) revealed that there was substantial molecular variation among individuals, accounting for 77.76% of the sample, with a significant *P*-value (<0.0001). The results suggest that genetic differences in *Ae. aegypti* originated primarily among individuals rather than among populations. Furthermore, the STRUCTURE and UPGMA cluster analyses showed that *Ae. aegypti* from the border areas were genetically isolated compared to those from the cities Ruili and Jinghong, consistent with the results of the Mantel test (*R* ^2^ = 0.245, *P* < 0.0001).

**Conclusions:**

Continuous invasion contributes to the maintenance of *Ae. aegypti* populations’ genetic diversity and different invasion accidents result in the genetic difference among *Ae. aegypti* populations of Yunnan Province.

**Electronic supplementary material:**

The online version of this article (doi:10.1186/s13071-017-2213-6) contains supplementary material, which is available to authorized users.

## Background

Dengue fever (DF) is an acute systemic viral disease caused by dengue virus and transmitted by *Aedes* mosquitoes. There are approximately 390 million dengue infections per year, and approximately 40% of the world population is at risk of infection from dengue [1]. Since 2013, local transmission of DF infections has been caused by imported cases of DF that have repeatedly occurred in Yunnan Province, especially in the cities of Ruili and Jinghong [2]. *Aedes aegypti* is the primary vector of the arboviruses, spreading dengue, yellow fever, chikungunya, Zika fever and other diseases [3–6] that originated in Africa and invaded the Americas and Southeast Asia through the shipping trade in the late nineteenth Century [7–9]. Previous studies have indicated that this species is distributed only in the area south of 22 degrees north latitude in China, which includes southern Taiwan, Hainan Island, and some sections of Guangdong and Guangxi Provinces, including islands [10].

Yunnan Province is located in southwestern China and extends from 21°8′32″ to 29°15′8″N and 97°31′39″ to 106°11′47″E. The climate in most regions of this province is fairly mild in winter and rather cool in summer. Because of its geographical position and natural climate conditions, Yunnan Province has the greatest diversity and abundance of mosquito species in China. However, there was no record of *Ae. aegypti* in Yunnan Province until 2002 [11].

As Yunnan Province is along the main passageway connecting South Asia and Southeast Asia, the international tourism industry as well as logistics and communications between Yunnan Province and neighbouring countries where *Ae. aegypti* is widespread [12], such as Myanmar, Cambodia, Vietnam and Thailand, have expanded in recent years. As previously reported, human trade and travel might support *Ae. aegypti* migration [13], and the invasion risk *via* human travel has become much more severe. Since the first report of *Ae. aegypti* in Yunnan Province at Ruili Jiegao Port, the species has been continuously found in nine cities in Yunnan Province [11, 14–18].

Biological invasions are closely associated with human health and genetic factors, such as genetic variation and admixture, and this information has been shown to be extremely useful for managing invasive species [19, 20]. Therefore, knowledge about the genetic diversity and structure among *Ae. aegypti* populations can help with inferring their invasion processes and with modelling the spread of disease and of insecticide resistance [21], which is useful for maintaining effective vector control strategies and providing insight into the epidemiology of arthropod-borne diseases such as DF [22–24]. Therefore, it is necessary to understand the invasion strategies, colonisation and dispersal of this mosquito species, especially from a population genetics perspective. However, to date, no studies have investigated the genetic diversity and structure of *Ae. aegypti* populations in Yunnan Province or even in China overall.

Microsatellites are short lengths of DNA sequences in which motifs of 1–6 bases are tandemly repeated, and they occur throughout the eukaryotic genome [25], providing many advantages, such as high polymorphism, co-dominant expression, and broad genome distribution [26]. Since the microsatellite markers for *Ae. aegypti* were isolated in 2001 [27], they have become a powerful tool that has been widely applied in population genetic studies of *Ae. aegypti*. Several studies on the genetic structure of *Ae. aegypti* using microsatellite markers have been conducted in Southeast Asia [28–34].

To explain the invasion, colonisation and dispersal of *Ae. aegypti* in Yunnan Province, we conducted a population genetics study of 28 locations in this area, using nine microsatellite loci. The results of this study can also provide a theoretical basis for programs for controlling *Ae. aegypti* in China.

## Methods

### Mosquito sampling

**Fig. 1:**
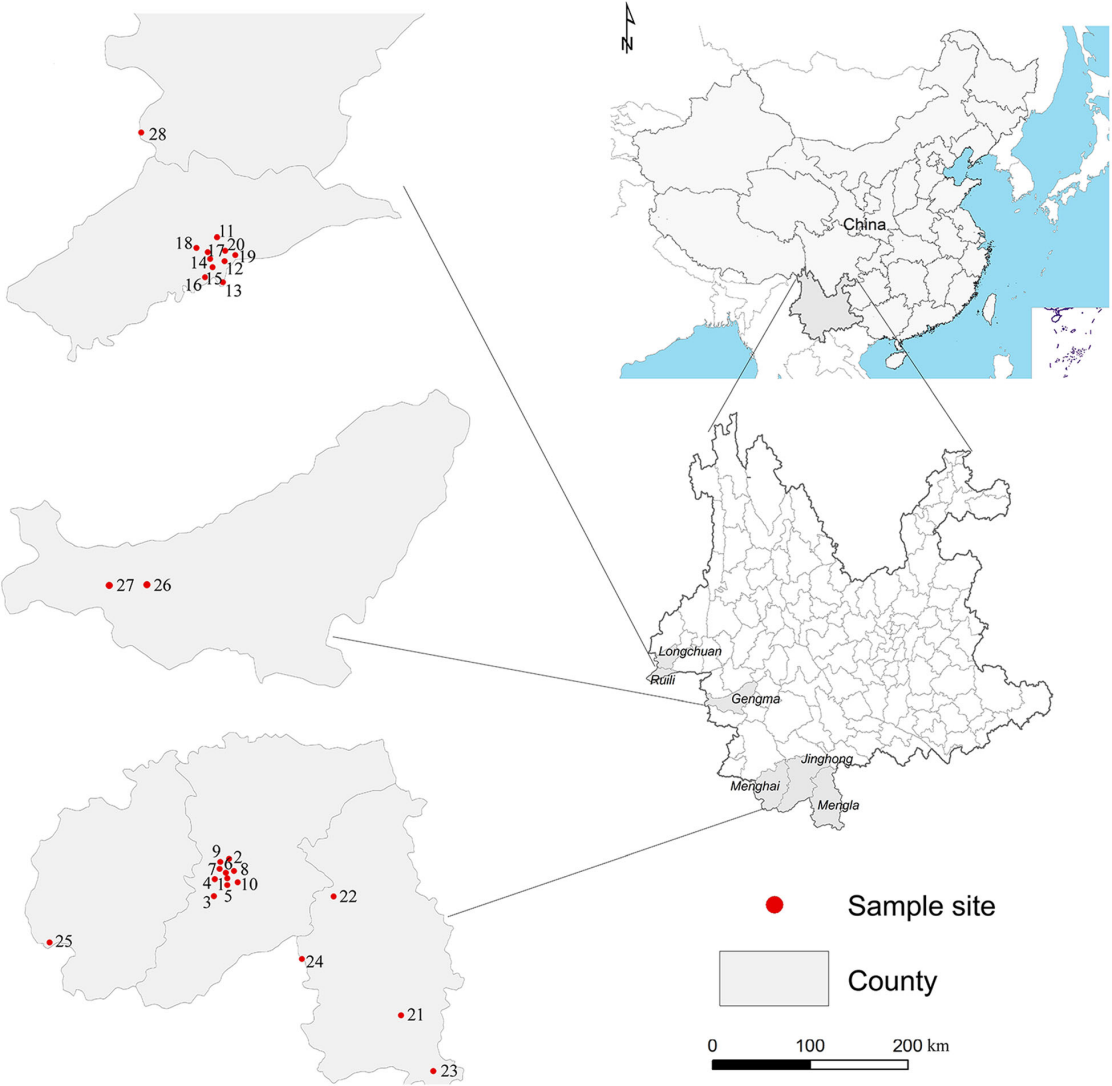
Illustration of the geographical locations of *Ae. aegypti* collection sites

Based on the results of previous investigations and the distribution of *Ae. aegypti* in Yunnan Province, 28 locations in Yunnan Province were chosen as research sites (Fig. 1, Table 1). All of the *Ae. aegypti* adults and larvae used in this study were collected from these regions from 2015 to 2016. The distances between the sampling sites ranged from several hundred meters (> 500 m) to kilometres. The precise locations of these sites were recorded using the global positioning system.

**Table 1:** Sampling information of Ae. aegypti collection in Yunnan Province,China

Collection region	No.	Location name (code)	No. of samples	Geographical coordinates	Collection date
Jinghong city	1	Linjiangyuan (LJY)	30	22°0′49 “N, 100°48′6”E	05/12/2015
	2	Nonken Hospital (NKH)	30	22°1′49″N, 100°47′35″E	05/15/2015
	3	Gasa town (GSZ)	30	21°57′5″N, 100°45′44″E	05/19/2015
	4	Damanyao (DMY)	30	22°0′0″N, 100°46′37″E	05/24/2015
	5	Guanguang Hotel (GGH)	30	22°0′12″N, 100°47′54″E	06/02/2015
	6	Galan Road (GLR)	30	22°1′8″N, 100°47′45″E	06/05/2015
	7	Ganxiusuo (GXS)	30	22°0′4″N, 100°47′24″E	06/07/2015
	8	Jiangbei (JBQ)	30	22°1′51″N, 100°47′15″E	06/12/2015
	9	Yunshu Company (YSC)	30	22°1′30″N, 100°47′15″E	06/15/2015
	10	Pi’er Park (PSS)	30	22°0′58″N, 100°49′3″E	06/17/2015
Ruili city	11	Munao Road (MNL)	30	24°1′38″N, 97°52′5″E	08/12/2015
	12	Jindian Road (JDL)	30	24°1′3″N, 97°52′56″E	08/15/2015
	13	Jiegao Freight Yard (JGH)	30	23°58′40″N, 97°53′24″E	08/19/2015
	14	Candy Company (TCP)	30	24°0′40″N, 97°52′42″E	08/22/2015
	15	Jincheng Steel Plant (JCG)	30	24°0′0″N, 97°52′18″E	08/24/2015
	16	Guomen bridge (GMG)	30	23°59′24″N, 97°52′3″E	08/27/2015
	17	Hongda Freight Yard (HDH)	30	24°0′52″N, 97°52′11″E	08/28/2015
	18	North Bus Terminal (BNS)	30	24°1′20″N, 97°51′35″E	08/30/2015
	19	Automobile Company (CXT)	30	24°00′53″N, 97°53′24″E	09/02/2015
	20	Scrapyard (BFC)	30	24°0′40″N, 97°52′12″E	09/05/2015
Border areas	21	Nanla Middle School (ML-1)	30	21°27′59″N, 101°34′2″E	05/10/2016
	22	Botanical Garden (ML-2)	30	21°56′24″N, 101°15′41″E	05/13/2016
	23	Shangyong (ML-3)	28	21°14′34″N, 101°42′44″E	05/15/2016
	24	Guanlei (ML-4)	30	21°40′47″N, 101°8′11″E	05/17/2016
	25	Daluo (MH-1)	25	21°42′6″N, 100°3′23″E	07/08/2016
	26	Mengding-1 (GM-1)	30	23°33′53″N, 99°5′48″E	07/12/2016
	27	Mengding-2 (GM-2)	30	23°33′00″N, 99°3′33″E	07/14/2016
	28	Longchuan (LC-1)	30	24°11′56″N, 97°43′36″E	07/20/2016

*Abbreviation*: *No* number of locations

A human-baited method with handheld aspirators performed according to the Surveillance Methods for Vector Density - Mosquito (GB/T 23797–2009), was used in the collection of adult mosquitoes (i.e. intercepted before biting), and field identification of the samples was performed through analysis of morphological characteristics [35]. To reduce the chance of collecting siblings, we selected only one mosquito per container if the samples were from larvae, and all larvae were reared to adulthood in the laboratory until they could be identified. All adult *Ae. aegypti* samples were preserved in 100% ethanol at 4 °C for the isolation of genomic DNA.

### DNA isolation, PCR amplification and microsatellite genotyping

In the present study, 30 *Ae. aegypti* samples per population were randomly selected from each site for genetic analyses. For sites with fewer than 30 mosquito samples, all the samples were selected. Genomic DNA was extracted individually from the samples using a TaKaRa Mini-BEST Universal Genomic DNA Extraction Kit (Takara, Dalian, China) and following the standard DNA extraction protocol provided by the manufacturer. The DNA yield was checked with the primers LCO1490 and HCO2198; in total, 833 samples were obtained and were stored at -20 °C until analysis.

Using denaturing polyacrylamide gel electrophoresis, nine microsatellite polymorphic loci were selected from 74 loci that were previously described in related studies [22, 26, 36]. The most suitable annealing temperature for each primer pair was confirmed with preliminary PCR amplifications, and the forward sequence of each primer was fluorescently labelled with FAM, HEX or TAMRA. The primer sequences and information are summarised in Table 2.

**Table 2:** Primer information for nine microsatellite loci screened in *Ae. aegypti* from Yunnan Province, China

Locus	Repeat motif	Primer sequence (5′-3′)	Annealing temperature (°C)	Allele size (bp)	Fluorescence labeling	Reference
SQM1	CT10(TT)CT	F: AATCGTGACGCGTCTTTTG; R: TAACTGCATCGAGGGAAACC	54	233–239	5′-HEX	[27]
SQM2	GA15	F: CAAACAACGAACTGCTCACG; R: TCGCAATTTCAACAGGTAGG	53	157–183	5′-FAM	[27]
SQM3	CAT7	F: ATTGGCGTGAGAACATTTTG; R: GAGGAGTGAGCAGATAGGAGTG	58	156–186	5′-FAM	[27]
SQM4	TAGA8	F: GCCAAAAACCAACAAACAGG; R: AATCGACCCGACCAATAACA	53	286–290	5′-TAMRA	[27]
SQM5	ATA36	F: GGAGCATTCATAGAGAATTGTCA; R: GAGATGAACCAGTCATAGGGC	56	110–116	5′-FAM	[27]
SQM6	(TTTA)7(T)14	F: CGACAGATGGTTACGGACGG; R: GTC CCG CTC CAA AAA TGC CC	58	228	5′-FAM	[23]
SQM7	AG4	F: AAAACCTGCGCAACAATCAT; R: AAGGACTCCGTATAATCGCAAC	56	147–169	5′-FAM	[36]
SQM8	AG5	F: TGATCTTGAGAAGGCATCCA; R: CGTTATCCTTTCATCACTTGTTTG	55	170–180	5′-FAM	[36]
SQM9	AC1	F: TCCGGTGGGTTAAGGATAGA; R: ACTTCACGCTCCAGCAATCT	55	193–209	5′-FAM	[36]

All PCR amplifications were conducted in a total volume of 20 μl using an S1000 Thermal Cycler (BIO-RAD, CA, USA). Each reaction contained 10 μl of 2× PCR Master Mix (Thermo Fisher Scientific, MA, USA), each primer at 0.8 μM, and 10 pg −1 μg of template DNA, with ddH_2_O, added to reach a volume of 20 μl. The PCR reaction conditions were as follows: 95 °C for 8 min, followed by 30 cycles at 94 °C for 45 s, a different annealing temperature for each locus (Table 2) for 30 s and 72 °C for 45 s. A final extension was conducted at 72 °C for 10 min. All the PCR products were diluted 1:10, mixed with LIZ 500-labeled size standard and formamide at a 1:9 ratio, and then run on an ABI3730XL (Applied Biosystems, Foster City, USA) capillary sequencer. Allele sizes for each locus were read with Gene Marker software (version 2.2.0).

### Microsatellite analysis

#### Genetic diversity within each population

To determine the genetic diversity within each sampling location, standard genetic parameters were calculated using several software programs. FSTAT (version 2.9.3.2) was used to assess allelic richness (r) in each population [37]. The inbreeding coefficient (F_IS_), observed heterozygosity (Ho) and expected heterozygosity (He) were calculated with Arlequin software (version 3.5.2.2) [38]. The linkage disequilibrium, departures from the Hardy-Weinberg equilibrium (HWE) and the *P*-value for each location were assessed using Fisher’s exact test in Gene pop (version 4.2) [39]. Null allele frequency for each microsatellite locus was estimated using Micro-checker software (version 2.2.3) [40]. The stepwise mutation model (SMM) were used to assess deviation from expected heterozygote excess related to allelic diversity across all nine loci and based on the loss of allelic diversity exceeding heterozygosity during a bottleneck effect in BOTTLENECK software (version 1.2.02) [41]. To determine whether the individuals in each location used in this study were sufficient for the research, the mean number of alleles per locus (N_A_) for each population was calculated with a rarefaction method by using the software Allelic Diversity Analyzer (version 1.0) [42]. *P*-values less than 0.05 were considered significant in each analysis. Polymorphic information content (PIC) across all nine loci was assessed using PIC-Calc 0.6 [43].

#### Population genetics structure and differences among populations

With regard to the analysis of the population genetics structure, the pair fixation indices (F_ST_) between populations and genetic distance [F_ST_/(1- F_ST_)] and the analysis of molecular variance (AMOVA) were performed with Arlequin (version 3.5.2.2). The software STRUCTURE (version 2.3.4) was used to assess the genetic structure differences among populations based on a Bayesian clustering approach with a Markov chain Monte Carlo algorithm (100,000 iterations and 1,000,000 repetitions). The *K* value was set from 1 to 31 (the true number of populations plus 3) with 20 runs for each *K* value [44, 45], and the *ΔK* methods were executed to determine the most likely *K* value [46]. A graphical display of the genetic structure was produced using DISTRUCT (version 1.1) [47]. To test the relationship between genetic distance [F_ST_/(1- F_ST_)] and geographic distance [ln (km)], a Mantel test with 10,000 permutations was performed using the ZT software package, and a graphical display of the results was produced with Excel 2016 (Microsoft Corporation, WA, USA) [48]. Based on the unweighted pair group method, UPGMA cluster analysis was conducted with the software NTSYS pc (version 2.2), and the polygenetic tree was modified with the software Tree View [49]. Principal component analysis (PCA) and non-metric multidimensional scaling (NMDS) analyses were conducted *via* the *Vegan* and *Permute* packages in R software [50].

## Results

### Genetic diversity

In total, 114 alleles were obtained for nine microsatellite loci. The highest number of alleles (20) was found at locus SQM6, and the lowest number (7) was found at locus SQM1. PIC was high, with values ranging from 0.392 to 0.886 (mean value 0.672). Micro-checker results suggest that null alleles were found at the loci SQM3, SQM4 and SQM9 and that the null allele frequency varied from 0 to 0.2956 among the 9 loci in all locations (Table 3). No consistency was found between any pair of loci across all locations, though 166 of 1008 (16.47%) pairwise tests for linkage disequilibrium remained significant after Bonferroni correction (Additional file 1: Figure S1).

**Table 3:** Null allele frequency, number of alleles and PIC information for genetic markers of *Ae. aegypti* from Yunnan Province, China

Locus	Number	Null allele frequency	Number of allele	PIC
SQM1	833	–	7	0.392
SQM 2	833	–	16	0.804
SQM 3	833	0.1703	13	0.629
SQM 4	833	0.2956	12	0.633
SQM 5	833	–	9	0.679
SQM 6	833	–	20	0.886
SQM 7	833	–	10	0.657
SQM 8	833	–	11	0.614
SQM9	833	0.1024	16	0.755
Mean			12.67	0.672

The genetic diversity and difference analysis of *Ae. aegypti* within 28 locations showed that the mean number of alleles per locus of each location ranged from 2.90 to 5.18, with an average of 4.04, and the allelic richness ranged from 3.34 to 5.72, with an average of 4.53. The average N_A_ value for Ruili City (5.15) was greater than in Jinghong City (4.88) and the border areas (4.63). The largest number of alleles (5.18) was observed at site HDH from Ruili City, while the lowest numbers were observed at M3 L (2.90) and M4 L (3.15) from the border areas. The He values for each location ranged from 0.353 to 0.681, and the Ho values for each location ranged from 0.401 to 0.689. The two locations M2 L and M3 L from the border areas had the highest and the lowest observed individual heterozygosity, respectively (M2 L, 0.689; M3 L, 0.401) (Additional file 2: Table S1).

The Hardy-Weinberg equilibrium (HWE) test revealed that only 2 of the 28 locations (7.14%) had significant departures from HWE, and the values of F_IS_ ranged from -0.147 to 0.157 (Additional file 2: Table S1). Among the three sampling areas, two locations from Ruili City had the highest mean allelic richness (JGH, 5.67; HDH, 5.72), and the lowest richness was observed in the border areas (M4 L, 3.67; M3 L, 3.34) (Additional file 3: Figure S2). The interaction analysis of the mean number of alleles per locus, Ho values and allelic richness of all locations also showed that the locations from Ruili City had higher genetic diversity than those from Jinghong City and the border areas. The SMM model analyses (Additional file 4: Table S2) also revealed that nearly 50% and 60% of sampling locations from the border areas and Ruili City showed significant population expansion, while all *P*-values (*P* > 0.05) remained non-significant among the populations of Jinghong City after Bonferroni correction.

### Genetic structure and differences

**Fig. 2:**
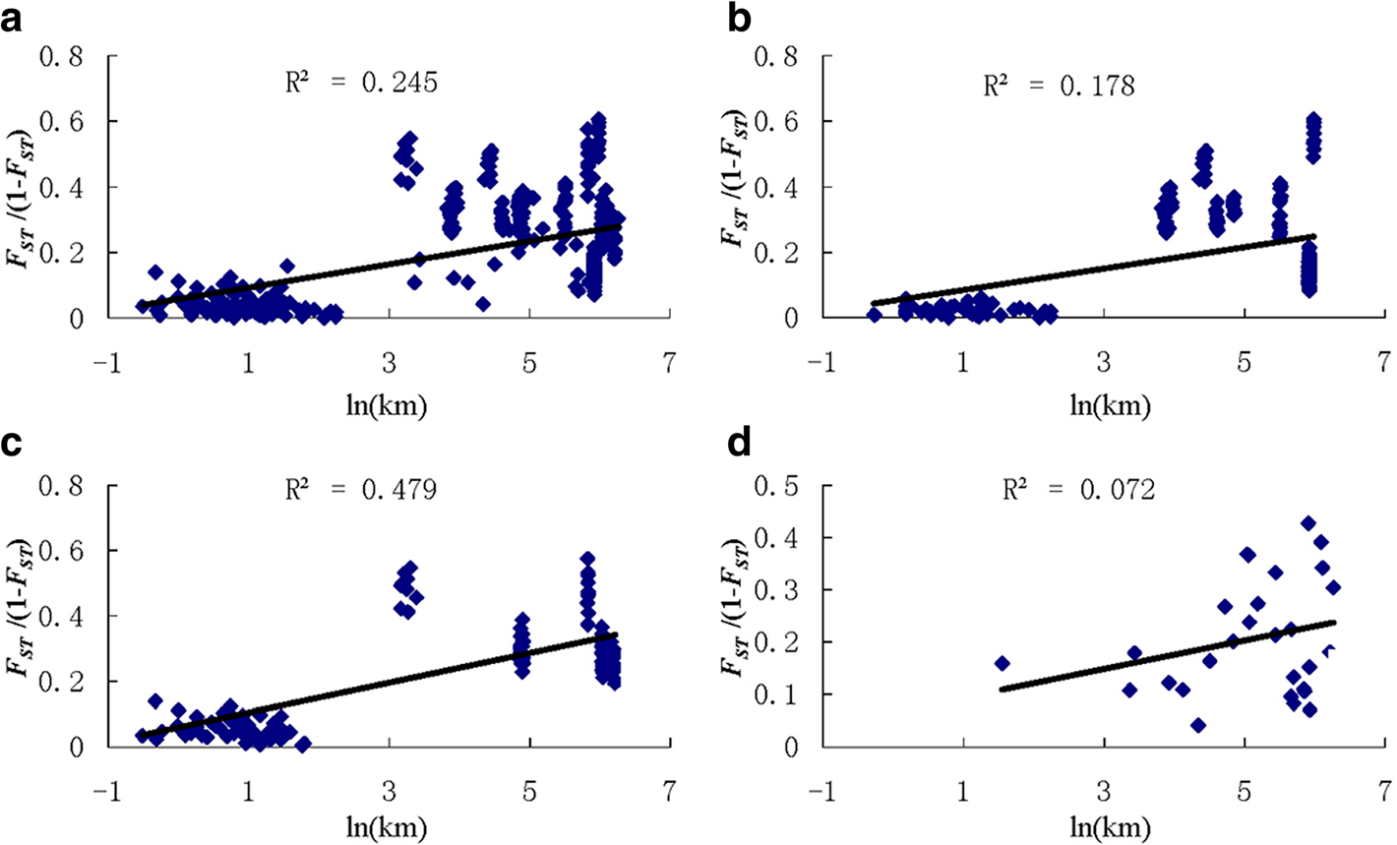
Analysis on the relationship between genetic distance [F_ST_/(1-F_ST_)] and geographical distance [ln(km)] for (**a**) all locations (*R* ^2^ = 0.245, *P* < 0.001); (**b**) locations from Jinghong city only (*R* ^2^ = 0.178, *P* < 0.001); (**c**) locations from Ruili city only (*R* ^2^ = 0.479, *P* < 0.001); and (**d**) locations from border area only (*R* ^2^ = 0.072, *P* = 0.167)

The Mantel test for the 28 locations showed a significant positive correlation (49.5%, *P <* 0.0001) between genetic distances and geographical distances. Using the isolation-by-distance model, the genetic distribution of all 28 locations was illustrated and is shown in Fig. 2a (*R* ^2^ = 0.245, *P* < 0.01). The genetic distances are positively correlated with geographical distances within the 10 locations in the cities Jinghong and Ruili (*R* ^2^ = 0.178 and 0.479, respectively; *P* < 0.01; Fig. 2b, c), but no such significant correlation was observed within the 8 locations of the border areas (*R* ^2^ = 0.072, *P* = 0.167; Fig. 2d).

The F_IT_ and F_CT_ values for *Ae. aegypti* were 0.222 and 0.145, respectively (both *P* < 0.01). This result indicates that genetic differences may exist mainly among individuals and groups. The AMOVA results (Additional file 5: Table S3) also indicated that the major variation in *Ae. aegypti* was mainly found among individuals and groups, accounting for 77.76% and 14.51% of the variation, respectively, while the variations among populations within groups and individuals within populations accounted for only 6.03% and 1.73%, respectively. Fisher’s exact tests showed that there was significant genetic variation in these four levels.

The pairwise F_ST_ values of *Ae. aegypti* ranged from -0.004 to 0.377, as displayed in Additional file 6: Table S4. Among these comparisons, 361 out of 378 (95.5%) showed significant genetic differences. The F_ST_ values were lower than 0.05 among 10 locations from Jinghong City, between 0.05 and 0.10 among 10 locations from Ruili City and above 0.10 among 8 locations from the border areas. All *P*-values were significant (*P* < 0.05) after Bonferroni corrections were applied.

**Fig. 3:**
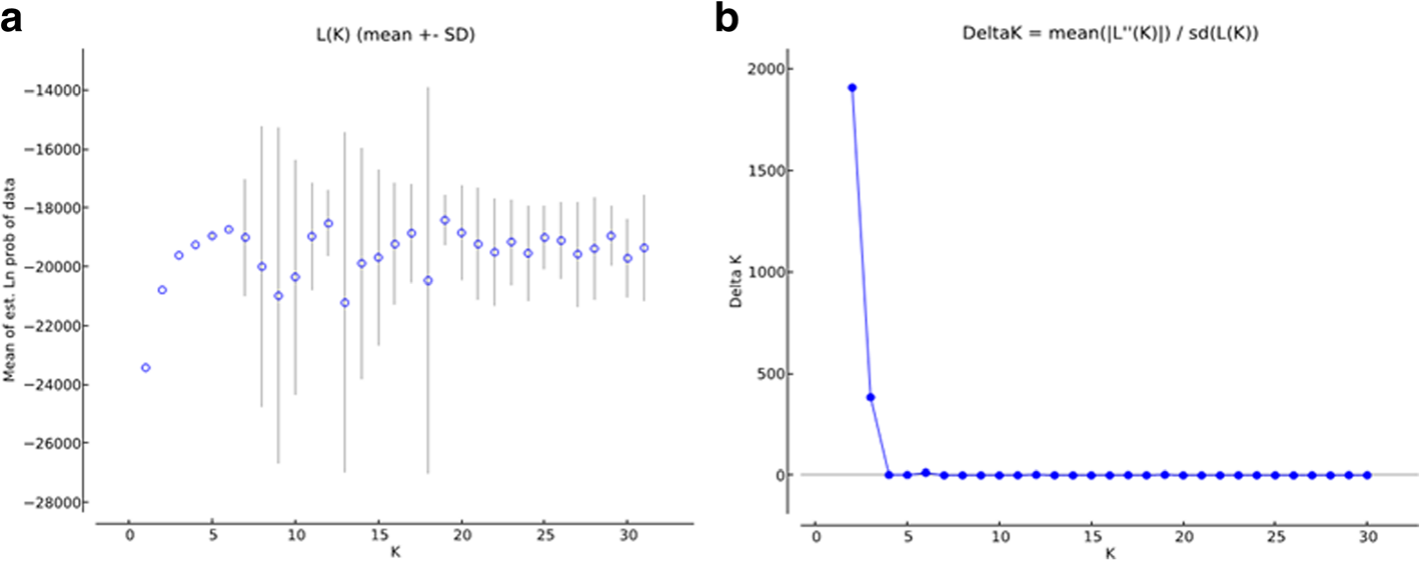
Scatter plots of Log probability of the data (**a**) and △K (**b**) for all *Ae. aegypti* populations analysed. Delta K plots are based on the rate of change in the log probability of the data between successive *K* values

**Fig. 4:**
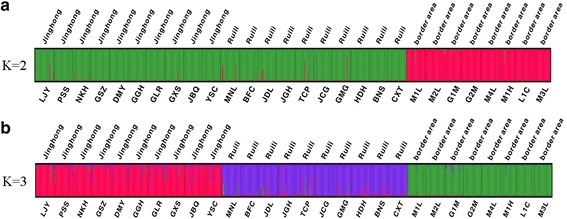
Genetic structure within 28 locations of *Ae. aegypti*. STRUCTURE bar plots are indicating relatedness of *Ae. aegypti* populations based on nine microsatellite loci. Each vertical bar represents an individual. The height of each bar represents the probability of assignment to each of Koptimal clusters (different colours) determined using Evanno et al.’s *ΔK* methods. **a** K = 2. **b** K = 3

**Fig. 5:**
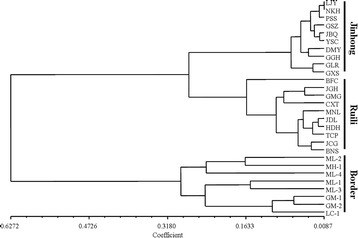
UPGMA cluster analysis of 28 sampling locations based on the genetic distance of all populations

**Fig. 6:**
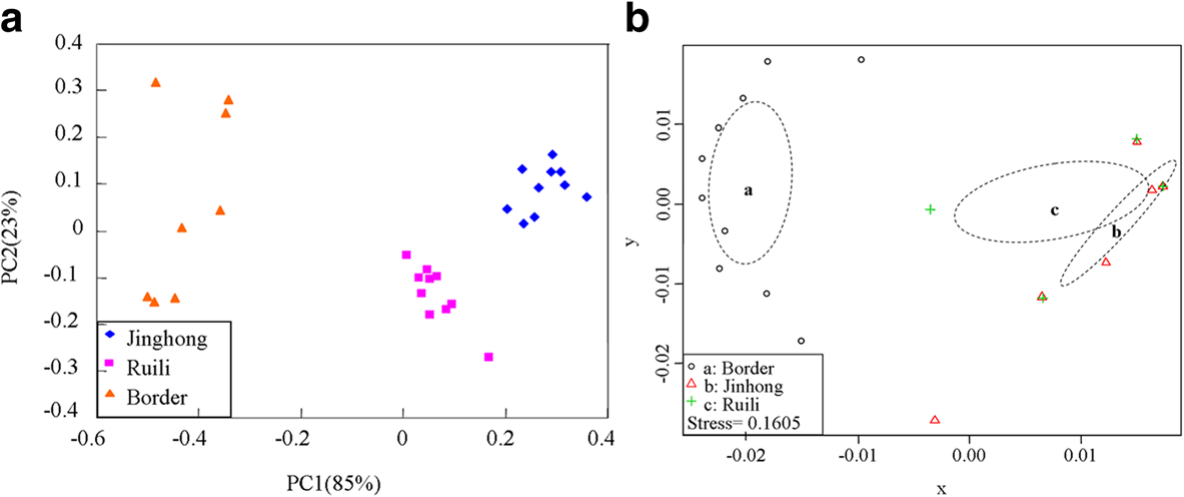
Correlation analysis among Ruili, Jinghong and the border sites. Principal coordinates analyses (**a** PCoA analysis) and non-metric multidimensional scaling (**b** NMDS analysis), based on co-dominant alleles at nine microsatellite loci, displaying genetic similarities among individuals of *Ae. aegypti* sampled from Ruili, Jinghong and the border sites

Based on Evanno et al.’s *ΔK* methods [44], all locations in this study were assigned to two genetically differentiated groups (K = 2, Fig. 3a, b) that overall showed a considerable mixture and that *Ae. aegypti* samples from the border areas were more genetically isolated compared to the two other sampling areas. Bayesian clustering analysis on all these collections also identified that the *Ae. aegypti* from the border areas were genetically isolated from Ruili and Jinghong cities (Fig. 4). Additionally, the UPGMA cluster analysis (Fig. 5) displayed similar results showing that 8 locations from the border areas clustered first and then grouped with 20 locations from Jinghong City and Ruili City, which were also separated into two different sub-clusters with strong support. PCA and NMDS analyses were illustrated in Fig. 6a, b. The results revealed that the genetic structures among the locations from the border areas were significantly different from the structures in Ruili and Jinghong.

## Discussion

Most species can be transported to a new geographical area *via* human activities but either fail to establish or maintain their population with minimal impact [51]. By definition, the progress of one exotic species to the level of a biological invasion involves a significant increase within its new territory [52]. *Aedes aegypti*, an exotic mosquito species in China was not discovered in Yunnan Province until it was first reported in Ruili in 2002. This species is now established in nine cities in Yunnan Province [11, 14–18, 53], and its distribution range and abundance have increased significantly. Thus, *Ae. aegypti* has become an invasive mosquito species in Yunnan Province.

Our results indicate that the *Ae. aegypti* populations in the border areas most likely came recently from neighbouring countries, and that continuous invasion still exists, which may explain the negative values of F_IS_ and why the values of Ho are higher than those of He [54–56]. Meanwhile, natural selection might explain the existence of a bottleneck effect in half of these locations.

Jinghong and Ruili, famous for their tourist and jewellery trading industries, are two major cities in Yunnan Province and are also closely connected with neighbouring countries where *Ae. aegypti* is widespread, such as Myanmar, Thailand and Vietnam [12]. Based on the invasion history, it is most likely that the invasion of Ruili started from neighbouring countries; nevertheless, although *Ae. aegypti* was first reported in Jinghong in 2013; it remains unclear whether the *Ae. aegypti* population also came from neighbouring countries or simply dispersed from Ruili or border areas because of the continuous tourist and business activities between these areas.

As the values of F_IS_ are all positive for the sampling locations of Jinghong and Ruili, and because the F_IS_ values of Jinghong locations are lower than those of Ruili locations, the difference may come from different invasion times of *Ae. aegypti* in these two cities, since the *Ae. aegypti* population was reported nearly 11 years earlier in Ruili than in Jinghong. This hypothesis can also be reached from the bottleneck effect analysis results, with a bottleneck effect observed in nearly 60% of the *Ae. aegypti* populations in Ruili and showed significant heterozygosity deficit for population expansion but in none of those in Jinghong.

Bayesian analysis revealed high genetic structure among the populations studied, especially between the *Ae. aegypti* population of the border area and the populations of Ruili and Jinghong cities. This segregation can also be observed in the UPGMA dendrogram, NMDS and PCA analysis. This may be because of the *Ae. aegypti* population of Ruili, Jinghong and the border area came from different invasion accidents, and their dispersal ability was limited by the short flight range of this mosquito.

Our analysis also suggests that *Ae. aegypti* may have been introduced to Jinghong from Ruili or that the original locations of the *Ae. aegypti* populations in these two cities are close together. This idea was supported by UPGMA clustering analysis, which indicates that the genetic distance between *Ae. aegypti* populations in the cities of Jinghong and Ruili is closer than that of the border area population. Furthermore, multiple invasions can help the allelic richness of invasive populations to recover, but the richness is usually lower than the original population [57, 58]. In this study, the allelic richness of the *Ae. aegypti* population in Jinghong is higher than that in border areas but lower than that in Ruili.

Passive dispersal is the most common way for this mosquito to spread in southwestern China, given that *Ae. aegypti* has a very close association with humans and shelters in indoor habitats [59] and has a very short flight range, usually limited to several hundred meters [60–62]. Results of isolation by distance analysis reveal that the genetic distances are positively correlated with geographical distances within the sampling locations of Jinghong and Ruili (*r* = 0.422 and 0.692, respectively; *P* < 0.01), while no such significant correlation was observed within the locations of the border areas (*r* = 0.269, *P* = 0.167). This may reflect the translocation of mosquito eggs and aquatic stages in containers as a result of human travel throughout the cities [63]. As for the border areas without natural barriers [64], *Ae. aegypti* may be introduced to these areas from bordering countries through active dispersal or population expansion, but the colonisation and dispersal time has not been long enough to cause genetic differentiation.

The genetic diversity and population structure of *Ae. aegypti* found in this study revealed a significant population expansion within Yunnan Province, which resulted in the similarity of *Ae. aegypti* population between Ruili and Jinghong cities. Based on UPGMA, NMDS and PCA analysis, our study also suggests that the *Ae. aegypti* population of Ruili, Jinghong and the border area came from different invasion accidents that we believe to be associated with passive dispersal, aided by human activities and transportation, representing the invasion risk of *Ae. aegypti* is still severe, and there is an ever-greater risk of arthropod-borne diseases such as DF.

## Conclusion

In conclusion, high genetic diversity and genetic structure were demonstrated among two *Ae. aegypti* populations from Yunnan Province. Continuous invasion, aided by human activities and transportation, appears to contribute to the maintenance of genetic diversity in *Ae. aegypti* in this region. The genetic difference among these two *Ae. aegypti* populations may have arisen from different invasion incidents with subsequent dispersal limited by the short flight range of this mosquito. Overall, the results of this study can provide a theoretical basis for programs for controlling *Ae. aegypti* in China.

## Additional files

Additional file 1: **Figure S1.** Analysis of linkage disequilibrium in 28 locations (TIFF 7443 kb)

Additional file 2: **Table S1.** Population statistics for *Ae. aegypti* investigated using nine microsatellite loci. (DOCX 17 kb)

Additional file 3: **Figure S2.** The mean number of distinct alleles per locus for 28 sample locations. (TIFF 1958 kb)

Additional file 4: **Table S2.** *Aedes aegypti* heterozygosity tests. (DOCX 15 kb)

Additional file 5: **Table S3.** Analysis of molecular variance of populations from Jinghong, Ruili and the border areas. (DOCX 15 kb)

Additional file 6: **Table S4.** Pairwise population differentiation estimates (F_ST_) and geographical distance [ln(km)] between all locations of *Ae. aegypti*. (DOCX 22 kb)

## Abbreviations

AMOVA: Analysis of molecular variation
DF: Dengue fever
R: Richness
F_CT_: Genetic differences among groups
F_IS_: Inbreeding coefficient
F_IT_: Global heterozygote deficit across of all populations
F_ST_: Genetic differences among populations
He: Expected heterozygosity
Ho: Observed heterozygosity
HWE: Hardy-Weinberg equilibrium
NA: Number of alleles per locus
NMDS: Non-metric multidimensional scaling
PCA: Principal components analysis
PIC: Polymorphic information content
SMM: Stepwise mutation model
TPM: Two-phase model
UPGMA: Unweighted pair-group method with arithmetic averaging
