# Supplementary figures and images for: The genetic diversity and population structure of domestic Aedes aegypti (Diptera: Culicidae) in Yunnan Province, southwestern China
Source: Parasit Vectors. 2017 Jun 13;10:292. doi: 10.1186/s13071-017-2213-6 (PMC5470206; doi:10.1186/s13071-017-2213-6)

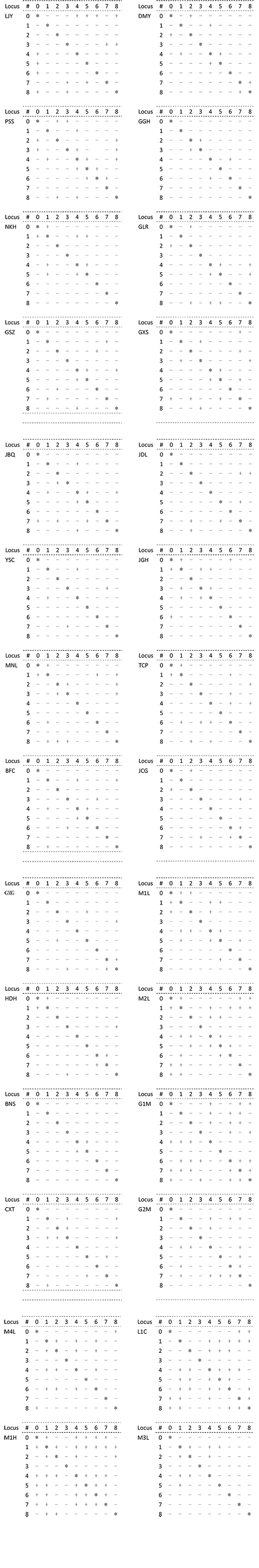

Supplement: Supplementary file 1 — Figure S1. Analysis of linkage disequilibrium in 28 locations (TIFF 7443 kb) [file 13071_2017_2213_MOESM1_ESM.tif]

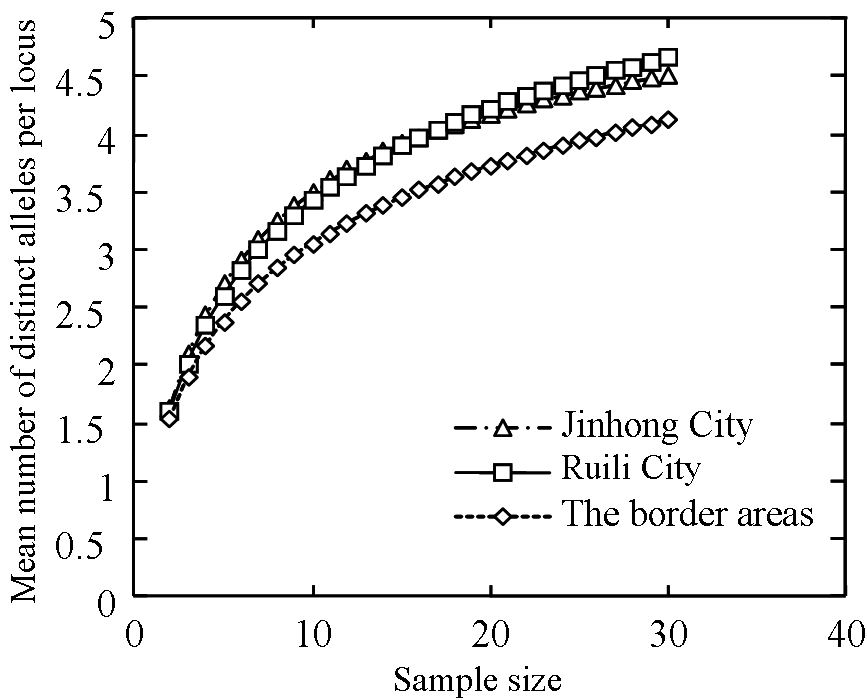

Supplement: Supplementary file 3 — Figure S2. The mean number of distinct alleles per locus for 28 sample locations. (TIFF 1958 kb) [file 13071_2017_2213_MOESM3_ESM.tif]
