# Supplementary material for: The genetic diversity and population structure of domestic Aedes aegypti (Diptera: Culicidae) in Yunnan Province, southwestern China
Source: Parasit Vectors. 2017 Jun 13;10:292. doi: 10.1186/s13071-017-2213-6 (PMC5470206; doi:10.1186/s13071-017-2213-6)
Supplement: Supplementary file 2 — Table S1. Population statistics for Ae. aegypti investigated using nine microsatellite loci. (DOCX 17 kb) [file 13071_2017_2213_MOESM2_ESM.docx]

**Table S1**  Population statistics for *Ae. aegypti* investigated using nine microsatellite loci

| Pop code | N | N_A_ | r | F_IS_ | Ho | He | HWE-*P* | | TPM | SMM |
| --- | --- | --- | --- | --- | --- | --- | --- | --- | --- | --- |
| LJY | 30 | 4.90 | 5.48 | 0.004 | 0.663 | 0.681 | | 0.111 | 0.21638 | 0.27825 |
| PSS | 30 | 4.81 | 5.34 | 0.095 | 0.611 | 0.680 | | 0.113 | 0.06195 | 0.27423 |
| NKH | 30 | 4.66 | 5.18 | 0.071 | 0.630 | 0.676 | | 0.258 | 0.47563 | 0.10615 |
| GSZ | 30 | 4.01 | 4.25 | 0.066 | 0.581 | 0.632 | | 0.178 | 0.06309 | 0.52520 |
| DMY | 30 | 4.58 | 4.97 | 0.073 | 0.615 | 0.656 | | 0.222 | 0.44688 | 0.47564 |
| GGH | 30 | 4.71 | 5.02 | 0.052 | 0.630 | 0.655 | | 0.199 | 0.18218 | 0.19587 |
| GLR | 30 | 4.46 | 4.77 | 0.092 | 0.622 | 0.646 | | **0.030** | 0.05051 | 0.44121 |
| GXS | 30 | 4.30 | 4.64 | 0.101 | 0.578 | 0.615 | | 0.150 | 0.41262 | 0.56594 |
| JBQ | 30 | 4.48 | 4.71 | 0.133 | 0.567 | 0.636 | | 0.114 | 0.44524 | 0.46590 |
| YSC | 30 | 4.16 | 4.39 | 0.064 | 0.567 | 0.604 | | 0.204 | 0.45374 | 0.52687 |
| MNL | 30 | 4.62 | 5.08 | 0.045 | 0.548 | 0.570 | | 0.215 | 0.54238 | **0.02624** |
| BFC | 30 | 3.99 | 4.25 | 0.120 | 0.556 | 0.628 | | 0.059 | 0.17570 | 0.42963 |
| JDL | 30 | 4.83 | 5.51 | 0.108 | 0.544 | 0.608 | | 0.261 | 0.47203 | **0.02986** |
| JGH | 30 | 5.04 | 5.67 | 0.078 | 0.544 | 0.588 | | 0.202 | 0.27681 | **0.02932** |
| TCP | 30 | 4.97 | 5.49 | 0.026 | 0.615 | 0.653 | | 0.246 | 0.21140 | 0.10404 |
| JCG | 30 | 3.77 | 4.11 | 0.128 | 0.456 | 0.525 | | 0.293 | 0.31486 | **0.03240** |
| GMG | 30 | 4.84 | 5.42 | 0.157 | 0.507 | 0.608 | | 0.234 | 0.45816 | **0.02686** |
| HDH | 30 | 5.18 | 5.72 | 0.077 | 0.596 | 0.658 | | 0.298 | 0.45211 | 0.10693 |
| BNS | 30 | 4.76 | 5.29 | 0.098 | 0.548 | 0.611 | | 0.223 | 0.26677 | 0.29500 |
| CXT | 30 | 4.55 | 4.91 | 0.138 | 0.530 | 0.622 | | 0.156 | 0.44714 | **0.02718** |
| M1L | 30 | 4.39 | 4.84 | -0.005 | 0.622 | 0.578 | | 0.188 | 0.41760 | 0.56625 |
| M2L | 30 | 4.12 | 4.45 | -0.136 | 0.689 | 0.604 | | 0.167 | 0.54295 | 0.26350 |
| G1M | 30 | 4.95 | 5.62 | 0.016 | 0.578 | 0.582 | | 0.184 | 0.10865 | **0.02684** |
| G2M | 30 | 4.16 | 4.54 | -0.147 | 0.652 | 0.572 | | 0.321 | 0.32148 | **0.03199** |
| M4L | 30 | 3.15 | 3.67 | -0.142 | 0.515 | 0.442 | | 0.371 | 0.39172 | 0.15817 |
| M1H | 25 | 4.61 | 5.22 | -0.029 | 0.618 | 0.605 | | **0.046** | 0.43038 | 0.41667 |
| L1C | 30 | 4.64 | 5.24 | -0.051 | 0.630 | 0.552 | | 0.066 | 0.56244 | **0.02662** |
| M3L | 28 | 2.90 | 3.34 | -0.065 | 0.401 | 0.353 | | 0.146 | 0.13494 | **0.02583** |

This table includes the genetic parameters: populations code, sample size (N), mean number of alleles per locus (N_A_), allelic richness (r), inbreeding coefficient (F_IS_), observed heterozygosity (Ho), excepted heterozygosity (He), *P*-values for Hardy-Weinberg equilibrium analysis (HWE-*P*), and *P*-values for heterozygote excess using two phase model (TPM) and stepwise mutation model (SMM). All *P*-values less than 0.05 were considered significant and given in bold.
