# Supplementary material for: The genetic diversity and population structure of domestic Aedes aegypti (Diptera: Culicidae) in Yunnan Province, southwestern China
Source: Parasit Vectors. 2017 Jun 13;10:292. doi: 10.1186/s13071-017-2213-6 (PMC5470206; doi:10.1186/s13071-017-2213-6)
Supplement: Supplementary file 4 — Table S2. Aedes aegypti heterozygosity tests. (DOCX 15 kb) [file 13071_2017_2213_MOESM4_ESM.docx]

**Table S2** *Aedes aegypti* heterozygosity tests.

| **Jinghong city** | | LJY | PSS | NKH | GSZ | DMY | GGH | GLR | GXS | JBQ | YSC |
| --- | --- | --- | --- | --- | --- | --- | --- | --- | --- | --- | --- |
| SMM | *He < Heq* | 5 | 5 | 6 | 4 | 3 | 2 | 3 | 4 | 3 | 4 |
|  | *He > Heq* | 4 | 4 | 3 | 5 | 6 | 7 | 6 | 5 | 6 | 5 |
|  | *P (He < Heq)* | 0.2783 | 0.2742 | 0.1062 | 0.5252 | 0.4756 | 0.1959 | 0.4412 | 0.5659 | 0.4659 | 0.5269 |

| **Ruili city** | | MNL | BFC | JDL | JGH | TCP | JCG | GMG | HDH | BNS | CXT |
| --- | --- | --- | --- | --- | --- | --- | --- | --- | --- | --- | --- |
| SMM | *He < Heq* | 7 | 3 | 7 | 7 | 6 | 7 | 7 | 6 | 5 | 7 |
|  | *He > Heq* | 2 | 6 | 2 | 2 | 3 | 2 | 2 | 3 | 4 | 2 |
|  | *P (He < Heq)* | **0.0262** | 0.4296 | **0.0299** | **0.0293** | 0.1040 | **0.0324** | **0.0267** | 0.1069 | 0.2950 | **0.0272** |

| **The border area** | | M1L | M2L | G1M | G2M | M4L | M1H | L1C | M3L |
| --- | --- | --- | --- | --- | --- | --- | --- | --- | --- |
| SMM | *He < Heq* | 4 | 5 | 7 | 7 | 6 | 4 | 7 | 6 |
|  | *He > Heq* | 5 | 4 | 2 | 2 | 3 | 4 | 2 | 1 |
|  | *P (He < Heq)* | 0.5666 | 0.2635 | **0.0268** | **0.0320** | 0.1582 | 0.4167 | **0.0266** | **0.0258** |

Number of loci exhibiting heterozygosity excess (*He*) and expected heterozygosity based on the number of observed alleles (*Heq*) under the SMM model. Significant *P*-values are in bold.
