# Supplementary material for: The genetic diversity and population structure of domestic Aedes aegypti (Diptera: Culicidae) in Yunnan Province, southwestern China
Source: Parasit Vectors. 2017 Jun 13;10:292. doi: 10.1186/s13071-017-2213-6 (PMC5470206; doi:10.1186/s13071-017-2213-6)
Supplement: Supplementary file 5 — Table S3. Analysis of molecular variance of populations from Jinghong, Ruili and the border areas. (DOCX 15 kb) [file 13071_2017_2213_MOESM5_ESM.docx]

**Table S3** Analysis of molecular variance of populations from Jinghong, Ruili and The Border areas

| Source of Variation | d. f. | Sum of Squares | Variance Components | Percentage of Variation | *P-* value | Fixation  indices |
| --- | --- | --- | --- | --- | --- | --- |
| Among groups | 2 | 565.877 | 0.48619 Va | 14.51 | *P* < 0.0001 | F_CT_ = 0.14506 |
| Among populations within groups | 25 | 367.478 | 0.20129 Vb | 6.01 | *P* < 0.0001 | F_SC_ = 0.07025 |
| Among individuals within populations | 805 | 2191.127 | 0.05783 Vc | 1.73 | *P* = 0.01075 | F_IS_ = 0.02171 |
| Within individuals | 833 | 2171.000 | 2.60624 Vd | 77.76 | *P* < 0.0001 | F_IT_ = 0.22238 |
| Total | 1665 | 5295.483 | 3.35155 |  |  |  |
