# Supplementary material for: The genetic diversity and population structure of domestic Aedes aegypti (Diptera: Culicidae) in Yunnan Province, southwestern China
Source: Parasit Vectors. 2017 Jun 13;10:292. doi: 10.1186/s13071-017-2213-6 (PMC5470206; doi:10.1186/s13071-017-2213-6)
Supplement: Supplementary file 6 — Table S4. Pairwise population differentiation estimates (FST) and geographical distance [ln(km)] between all locations of Ae. aegypti. (DOCX 22 kb) [file 13071_2017_2213_MOESM6_ESM.docx]

**Table S4** Pairwise population differentiation estimates (F_ST_) (below the diagonal) and geographical distance [ln(km)] (above the diagonal) between all populations of *Ae. aegypti*

| pop | LJY | NKH | GSZ | DMY | GGH | GLR | GXS | JBQ | YSC | PSS | MNL | JDL | JGH | TCP | JCG | GMG | HDH | BNS | CXT | BFC | ML-1 | ML-2 | ML-3 | ML-4 | MH-1 | GM-1 | GM-2 | LC-1 |
| --- | --- | --- | --- | --- | --- | --- | --- | --- | --- | --- | --- | --- | --- | --- | --- | --- | --- | --- | --- | --- | --- | --- | --- | --- | --- | --- | --- | --- |
| LJY |  | 0.788 | 2.079 | 1.099 | 0.182 | -0.261 | 0.693 | 0.47 | 0.693 | 0.531 | 5.927 | 5.922 | 5.913 | 5.922 | 5.922 | 5.92 | 5.924 | 5.927 | 5.92 | 5.921 | 4.603 | 3.859 | 4.846 | 3.926 | 4.437 | 5.504 | 5.511 | 5.986 |
| NKH | **-**0.002 |  | 2.241 | 1.335 | 1.163 | 0.405 | 1.253 | 0.262 | -0.274 | 1.194 | 5.921 | 5.916 | 5.908 | 5.916 | 5.916 | 5.915 | 5.918 | 5.922 | 5.915 | 5.916 | 4.624 | 3.888 | 4.862 | 3.968 | 4.437 | 5.495 | 5.502 | 5.981 |
| GSZ | -0.004 | 0.003 |  | 1.723 | 1.932 | 2.104 | 1.808 | 2.241 | 2.163 | 2.208 | 5.93 | 5.925 | 5.916 | 5.925 | 5.924 | 5.923 | 5.926 | 5.93 | 5.923 | 5.924 | 4.597 | 3.926 | 4.835 | 3.894 | 4.359 | 5.513 | 5.519 | 5.989 |
| DMY | **0.016** | **0**.010 | **0.023** |  | 0.833 | 1.065 | 0.182 | 1.411 | 1.131 | 1.526 | 5.924 | 5.919 | 5.91 | 5.919 | 5.918 | 5.917 | 5.921 | 5.925 | 5.917 | 5.918 | 4.615 | 3.906 | 4.853 | 3.942 | 4.402 | 5.501 | 5.508 | 5.983 |
| GGH | **0.019** | **0.014** | **0.024** | **0.033** |  | 0.531 | 0.182 | 0.993 | 1.03 | 0.875 | 5.928 | 5.923 | 5.914 | 5.923 | 5.923 | 5.922 | 5.925 | 5.929 | 5.922 | 5.923 | 4.599 | 3.861 | 4.841 | 3.914 | 4.428 | 5.507 | 5.514 | 5.987 |
| GLR | 0.008 | **0.017** | 0.009 | **0.047** | **0.013** |  | 0.788 | 0.182 | 0.182 | 0.788 | 5.925 | 5.92 | 5.911 | 5.92 | 5.919 | 5.918 | 5.922 | 5.926 | 5.918 | 5.919 | 4.611 | 3.871 | 4.852 | 3.944 | 4.434 | 5.501 | 5.508 | 5.984 |
| GXS | **0.037** | **0.045** | **0.030** | **0.056** | **0.033** | **0.022** |  | 1.224 | 1.03 | 1.253 | 5.926 | 5.921 | 5.912 | 5.921 | 5.921 | 5.92 | 5.923 | 5.927 | 5.92 | 5.921 | 4.606 | 3.882 | 4.847 | 3.926 | 4.415 | 5.504 | 5.511 | 5.989 |
| JBQ | **0.027** | **0.034** | **0.019** | **0.041** | **0.030** | **0.017** | 0.011 |  | 0.531 | 0.693 | 5.925 | 5.92 | 5.911 | 5.92 | 5.919 | 5.918 | 5.922 | 5.926 | 5.918 | 5.919 | 4.612 | 3.861 | 4.853 | 3.947 | 4.447 | 5.500 | 5.507 | 5.984 |
| YSC | **0.010** | 0.009 | **0.019** | 0.011 | **0.020** | 0.009 | **0.034** | **0.019** |  | 1.224 | 5.922 | 5.916 | 5.908 | 5.916 | 5.916 | 5.915 | 5.918 | 5.922 | 5.915 | 5.916 | 4.624 | 3.894 | 4.861 | 3.967 | 4.428 | 5.496 | 5.503 | 5.981 |
| PSS | 0.007 | 0.007 | 0.012 | 0.006 | **0.034** | **0.026** | **0.056** | **0.027** | 0.003 |  | 5.93 | 5.925 | 5.916 | 5.925 | 5.925 | 5.924 | 5.927 | 5.931 | 5.923 | 5.925 | 4.592 | 3.824 | 4.837 | 3.908 | 4.457 | 5.508 | 5.516 | 5.989 |
| MNL | **0.132** | **0.136** | **0.142** | **0.137** | **0.129** | **0.127** | **0.115** | **0.119** | **0.108** | **0.128** |  | 0.642 | 1.792 | 0.742 | 1.131 | 1.435 | 0.470 | 0.095 | 0.916 | 0.788 | 6.163 | 6.035 | 6.218 | 6.052 | 5.837 | 4.910 | 4.887 | 3.165 |
| JDL | **0.131** | **0.133** | **0.127** | **0.143** | **0.157** | **0.136** | **0.142** | **0.164** | **0.132** | **0.134** | **0.093** |  | 1.459 | -0.329 | 0.742 | 1.163 | 0.000 | 0.833 | -0.236 | -0.01 | 6.159 | 6.031 | 6.215 | 6.048 | 5.831 | 4.898 | 4.873 | 3.239 |
| JGH | **0.112** | **0.114** | **0.124** | **0.102** | **0.113** | **0.123** | **0.112** | **0.113** | **0.094** | **0.110** | **0.012** | **0.085** |  | 1.361 | 1.163 | 1.03 | 1.504 | 1.758 | 1.459 | 1.589 | 6.152 | 6.023 | 6.208 | 6.04 | 5.82 | 4.879 | 4.854 | 3.388 |
| TCP | **0.134** | **0.127** | **0.157** | **0.121** | **0.142** | **0.161** | **0.175** | **0.179** | **0.137** | **0.141** | **0.111** | **0.123** | **0.068** |  | 0.262 | 0.916 | -0.315 | 0.788 | 0.262 | 0.531 | 6.159 | 6.03 | 6.215 | 6.048 | 5.83 | 4.899 | 4.874 | 3.25 |
| JCG | **0.100** | **0.104** | **0.114** | **0.101** | **0.114** | **0.123** | **0.118** | **0.115** | **0.098** | **0.110** | **0.030** | **0.075** | 0.008 | **0.059** |  | 0.182 | 0.405 | 0.956 | 0.956 | 1.099 | 6.159 | 6.03 | 6.214 | 6.048 | 5.829 | 4.9 | 4.876 | 3.27 |
| GMG | **0.140** | **0.146** | **0.153** | **0.133** | **0.142** | **0.149** | **0.136** | **0.151** | **0.125** | **0.140** | **0.030** | **0.089** | **0.022** | **0.086** | **0.041** |  | 0.993 | 1.281 | 1.308 | 1.411 | 6.158 | 6.029 | 6.213 | 6.046 | 5.827 | 4.899 | 4.874 | 3.3 |
| HDH | **0.122** | **0.117** | **0.145** | **0.115** | **0.127** | **0.146** | **0.151** | **0.150** | **0.123** | **0.128** | **0.070** | **0.100** | **0.045** | **0.024** | **0.030** | **0.064** |  | 0.336 | 0.588 | 0.693 | 6.161 | 6.032 | 6.216 | 6.049 | 5.832 | 4.904 | 4.88 | 3.223 |
| BNS | **0.088** | **0.085** | **0.098** | **0.076** | **0.099** | **0.111** | **0.105** | **0.110** | **0.081** | **0.086** | **0.036** | **0.071** | 0.005 | **0.040** | **0.012** | **0.044** | **0.036** |  | 1.099 | 1.065 | 6.164 | 6.036 | 6.219 | 6.053 | 5.837 | 4.914 | 4.89 | 3.17 |
| CXT | **0.103** | **0.112** | **0.115** | **0.112** | **0.121** | **0.108** | **0.095** | **0.123** | **0.096** | **0.118** | **0.039** | **0.046** | **0.023** | **0.085** | **0.035** | **0.021** | **0.056** | **0.038** |  | -0.511 | 6.158 | 6.029 | 6.213 | 6.047 | 5.83 | 4.893 | 4.868 | 3.258 |
| BFC | **0.133** | **0.131** | **0.139** | **0.113** | **0.158** | **0.160** | **0.144** | **0.154** | **0.131** | **0.135** | **0.082** | **0.060** | **0.045** | **0.061** | **0.030** | **0.062** | **0.032** | **0.031** | **0.034** |  | 6.159 | 6.03 | 6.214 | 6.048 | 5.831 | 4.894 | 4.87 | 3.239 |
| ML-1 | **0.218** | **0.211** | **0.223** | **0.230** | **0.243** | **0.234** | **0.246** | **0.261** | **0.225** | **0.248** | **0.226** | **0.212** | **0.200** | **0.217** | **0.184** | **0.243** | **0.225** | **0.191** | **0.197** | **0.203** |  | 4.121 | 3.364 | 3.922 | 5.066 | 5.843 | 5.849 | 6.21 |
| ML-2 | **0.215** | **0.206** | **0.215** | **0.215** | **0.218** | **0.236** | **0.219** | **0.241** | **0.225** | **0.251** | **0.243** | **0.256** | **0.223** | **0.236** | **0.190** | **0.268** | **0.221** | **0.198** | **0.234** | **0.207** | **0.098** |  | 4.504 | 3.434 | 4.84 | 5.656 | 5.663 | 6.087 |
| ML-3 | **0.239** | **0.243** | **0.254** | **0.239** | **0.259** | **0.270** | **0.259** | **0.270** | **0.247** | **0.263** | **0.197** | **0.228** | **0.171** | **0.199** | **0.162** | **0.217** | **0.195** | **0.169** | **0.199** | **0.191** | **0.097** | **0.141** |  | 4.34 | 5.186 | 5.921 | 5.926 | 6.263 |
| ML-4 | **0.255** | **0.252** | **0.267** | **0.254** | **0.262** | **0.275** | **0.267** | **0.285** | **0.261** | **0.282** | **0.217** | **0.237** | **0.184** | **0.220** | **0.175** | **0.240** | **0.204** | **0.183** | **0.207** | **0.200** | **0.109** | **0.152** | **0.041** |  | 4.718 | 5.692 | 5.698 | 6.105 |
| MH-1 | **0.305** | **0.294** | **0.297** | **0.320** | **0.316** | **0.336** | **0.328** | **0.327** | **0.334** | **0.338** | **0.321** | **0.347** | **0.306** | **0.344** | **0.272** | **0.365** | **0.318** | **0.291** | **0.334** | **0.316** | **0.192** | **0.167** | **0.214** | **0.211** |  | 5.437 | 5.438 | 5.903 |
| GM-1 | **0.204** | **0.198** | **0.207** | **0.223** | **0.217** | **0.225** | **0.223** | **0.222** | **0.222** | **0.238** | **0.243** | **0.257** | **0.217** | **0.245** | **0.186** | **0.280** | **0.214** | **0.203** | **0.234** | **0.211** | **0.099** | **0.088** | **0.132** | **0.117** | **0.176** |  | 1.548 | 5.053 |
| GM-2 | **0.263** | **0.261** | **0.271** | **0.267** | **0.287** | **0.283** | **0.291** | **0.291** | **0.265** | **0.285** | **0.240** | **0.252** | **0.211** | **0.237** | **0.204** | **0.266** | **0.233** | **0.207** | **0.231** | **0.224** | **0.096** | **0.183** | **0.066** | **0.076** | **0.250** | **0.137** |  | 5.034 |
| LC-1 | **0.340** | **0.330** | **0.351** | **0.347** | **0.368** | **0.369** | **0.373** | **0.377** | **0.347** | **0.360** | **0.330** | **0.348** | **0.313** | **0.325** | **0.292** | **0.354** | **0.347** | **0.297** | **0.339** | **0.338** | **0.153** | **0.281** | **0.233** | **0.255** | **0.299** | **0.268** | **0.269** |  |

The significances were tested for multi comparisons by the Bonferroni method, *p* < 0.05; bolding displayed below the diagonal means significant difference; F_ST_ averaging between all populations displayed below the diagonal; geographical distance [ln(km)] displayed above the diagonal
